# A two-generation hyperparathyroidism-jaw tumor (HPT-JT) syndrome family: clinical presentations, pathological characteristics and genetic analysis: a case report

**DOI:** 10.1186/s13000-022-01248-x

**Published:** 2022-09-24

**Authors:** Dun Yang, Jiaoyun Zheng, Fei Tang, Qiongzhi He, Hui Huang, Peng Zhou

**Affiliations:** 1 Department of Pathology, Taoyuan People’s Hospital, Changde, China; 2 Department of Pathology, The Second Xiangya Hospital, Central South University, Changsha, China; 3 Department of Radiology, The Second Xiangya Hospital, Central South University, Changsha, China; 4 Geneplus-Beijing, Changsha, China; 5 Department of Medical Genetics, The Second Xiangya Hospital, Central South University, Changsha, China

**Keywords:** HPT-JT, Heritable jaw OF, Genetic testing, CDC73, MEST

## Abstract

**Background:**

Hyperparathyroidism-Jaw Tumor (HPT-JT) is caused by inactivating germline mutations of CDC73. This hereditary disease can present with a range of symptoms. Jaw ossifying fibroma (OF) is one of the most important clinical presentations, affecting 30% of HPT-JT patients. However, OF is easily confused with other fibro-osseous lesions (FOLs) of the jaw. The correct diagnosis of HPT-JT is a real challenge and must be confirmed by genetic testing.

**Case presentation:**

A female proband and her father suffered from multiple and recurrent FOLs in the jaw. Considering well demarcated margin and heterogeneous calcified substance lying in a variable density of fibrous stroma, we reached the diagnosis of jaw OF through radiologic and microscopic analyses. Additionally, the proband presented with chronic anemia resulting from menorrhagia, as well as renal mixed epithelial and stromal tumor (MEST). Two patients both presented with no evidence of Hyperparathyroidism (HPT). A germline start codon mutation (c.1A > G) of CDC73 was identified in them. Copy number loss at the CDC73 gene locus was verified in the jaw tumor sample of the proband.

**Conclusion:**

Regardless of whether HPT manifestations are present, patients with heritable jaw OF may be at risk for HPT-JT. Genetic testing should be adopted to confirm the diagnosis. Early recognition of HPT-JT helps to better develop tailored treatment plans and surveillance programs.

## Background

Hyperparathyroidism-Jaw Tumor (HPT-JT) is an autosomal-dominant inherited syndrome with incomplete penetrance and variable expression, caused by germline mutations of the CDC73 [1]. The most common manifestation of HPT-JT is hyperparathyroidism (HPT), resulting from parathyroid benign adenoma or malignant carcinoma. Jaw ossifying fibroma (OF) and kidney lesions may also occur in about 30 and 15% of HPT-JT patients respectively [2, 3]. In addition, some researchers have reported that female patients developed uterine tumours at the penetrance rate of 50–75% [2–5]. Parafibromin, encoded by CDC73 gene, exhibits antiproliferative properties by acting as a part of Polymerase Associated Factor 1 complex [6, 7], directly interacting with nuclear b-catenin and Gli proteins [8, 9], and downregulating cyclin D1 [10]. Loss of parafibromin expression resulting from variable inactivated CDC73 mutations have been reported in HPT-JT related tumors, giving rise to tumorigenesis [5, 11–14]. Among different CDC73 germline mutations, over 75% mutations are frameshift mutations and nonsense mutations, resulting in either the truncation of the parafibromin protein or loss of the translated protein through nonsense-mediated mRNA decay [15].

Commonly, fibro-osseous lesions (FOLs) in the maxillofacial region include osseous dysplasia (OD), fibrous dysplasia (FD) and OF [16]. As the most commonly encountered FOLs of the jaws in clinical practice, OD is referred to as cemental dysplasia, cemento-osseous dysplasia or cementoma. FD is a common benign skeletal lesion resulting from a failure in the remodelling process of immature bone to mature lamellar bone, with a predilection for the long bones, ribs, and craniofacial bones [17]. Polyostotic FD may be a clinical presentation of McCune-Albright Syndrome (MAS), which is characterized by cafe-au-lait skin lesions and hyperfunctioning endocrinopathies as well [18].. OF is a benign tumor which thought to arise from the periodontal ligament [19], and can occur predominantly in the molar and premolar regions of the mandible [16]. Distinguishing among OD, FD and OF is a great pathological challenge because of their histological similarity. The correct diagnosis of FOLs in the jaw relies on careful correlations among clinical presentation, radiographic appearance and histopathological features [16, 20]. All three kinds of disorders may not only present with polyostotic bone lesions, but also as a part of tumour syndromes, especially hereditary syndromes [17]. Herein, we report a two-generation HPT-JT syndrome family, presenting with multiple/recurrent hereditary jaw OF. Germline and somatic mutation testing, pathological and clinical observation, and radiographical analysis were displayed comprehensively.

In HPT-JT, although the same CDC73 germline mutation, no apparent genotype–phenotype correlation was discovered [15]. Moreover, nonpenetrance is observed in > 30% of CDC73 mutation carriers [4]. Based on the incomplete penetrance and variable expression, as well as the scarcity, recognizing hereditary disorders related to CDC73 germline mutation is difficult. As the primary clinical feature, presence of HPT is reminiscent of HPT-JT syndrome. On the contrary, absence of HPT may result in missed diagnosis of HPT-JT syndrome. Our reported patients in this family both have no HPT.

## Case presentation

**Fig. 1:**
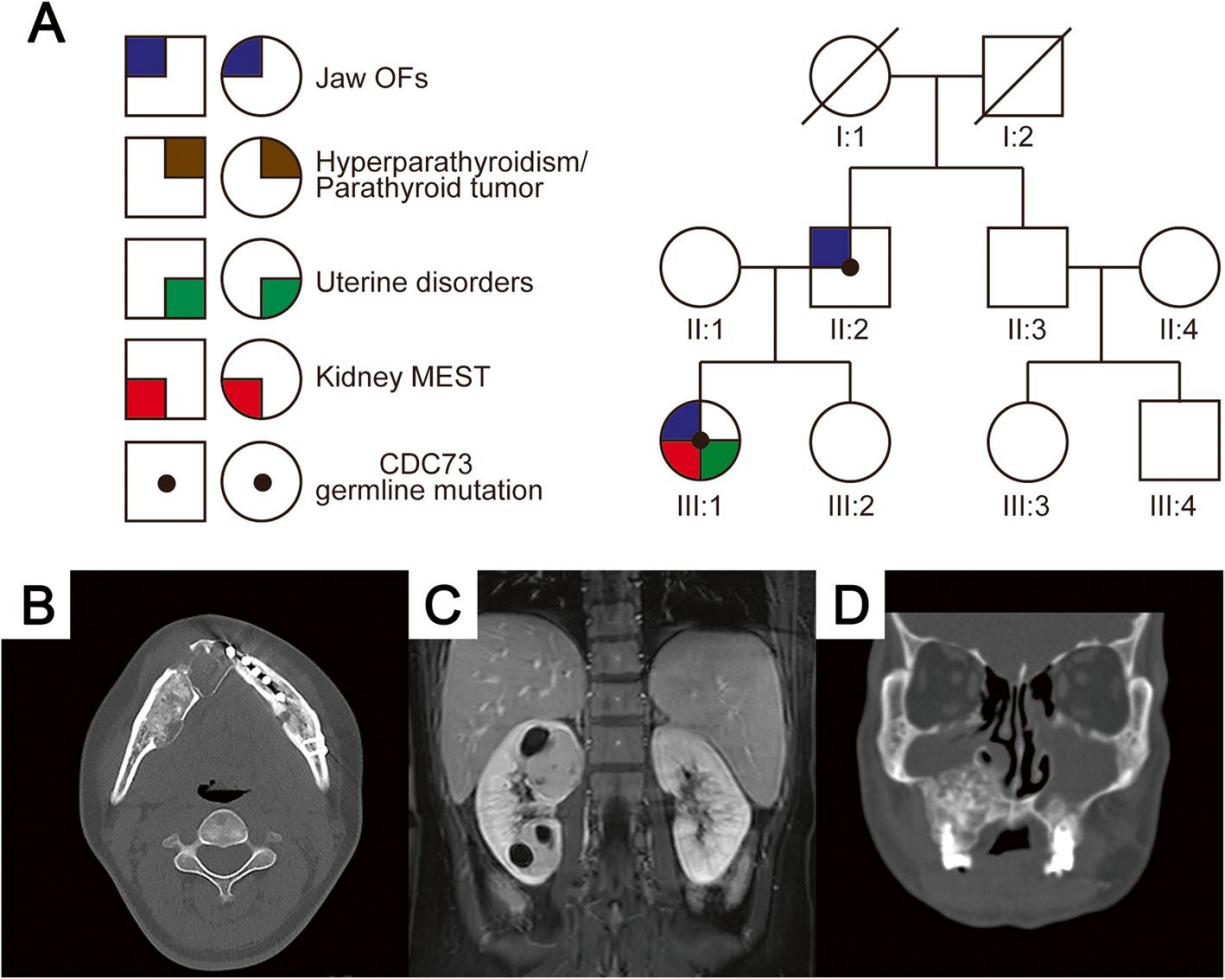
Family pedigree and imaging. **A** Patients are designated by generation (Roman numerals I, II, III) and individual (Arabic numerals 1, 2, 3, 4). CDC73 germline mutation carriers are denoted with central black dots. Clinical manifestations related to CDC73 germline mutation are indicated in the quadrants as shown. **B** Axial CT of the patient III:1 showing multiple mixed-density lesions in the bilateral mandible, which are expansile and well demarcated. In the right mandible, central radiopacities are partly surrounded by a thin radiolucent rim. These lesions displaced teeth and the border of the mandible. A denovo lesion of the right mandible near the centerline showing low density. **C** Coronal MRI imaging of patient III:1 demonstrated two cystic/solid leisions in the right kidney showing mild enhancement during the delayed phase. **D** Coronal CT scan of the patient II:2 demonstrating bilateral maxillae lesions with mixed density. The right lesion is more obvious, invasion of the right maxillary sinus

The proband (III:1, Fig. 1A) was a 32-year-old nulligravida woman who presented with recurrent and bilateral multiple FOLs in the mandible (Fig. 1B). She had undergone six surgeries, causing disfigurement of the facial features and teeth deformities. She had noticed a mass in the left mandible when she was 11 years old, and undergone a tumorectomy. Currently, at admission to our hospital, she also had a 2-year history of two cystic/solid tumors (4.1 × 5.2 cm and 4.9 × 4.6 cm on MRI scan) in the right kidney, which was classified as a Bosniak III or IV lesion, indicating a pre-operative clinical impression of cystic renal cancer (Fig. 1C). She accepted percutaneous renal puncture surgery. Additionally, she had suffered from life-long menorrhagia resulting in anemia. Ultrasonography of the urinary and genital system showed leiomyomas and endometrial hyperplasia (results of out-of-hospital are not shown). The proband did not present with polydipsia, polyuria, nocturia, constipation and hematuresis. Biochemical analysis results: normocalcemia (2.23 mmol/L, reference range: 2.11–2.52 mmol/L), normal levels of intact serum parathyroid hormone (25 pg/mL, reference range: 18.5–88 pg/mL), hypohemoglobinemia (70 g/L, reference range: 130–175 g/L). A technetium 99 m sestamibi parathyroid scan revealed no parathyroid tumor.

The patient’s father, II:2, also had undergone a tumorectomy when he was 11 years old on account of FOLs in the mandible. He had experienced total mandibulectomy at age 44 because of multiple-recurrent lesions in the mandible. Upon presentation to our hospital for evaluation at age 56, de novo FOLs were identified in the bilateral maxillae, especially in the right (Fig. 1D). He had not presented with HPT over the years. Blood routine examination and renal ultrasonography are normal.

**Fig. 2:**
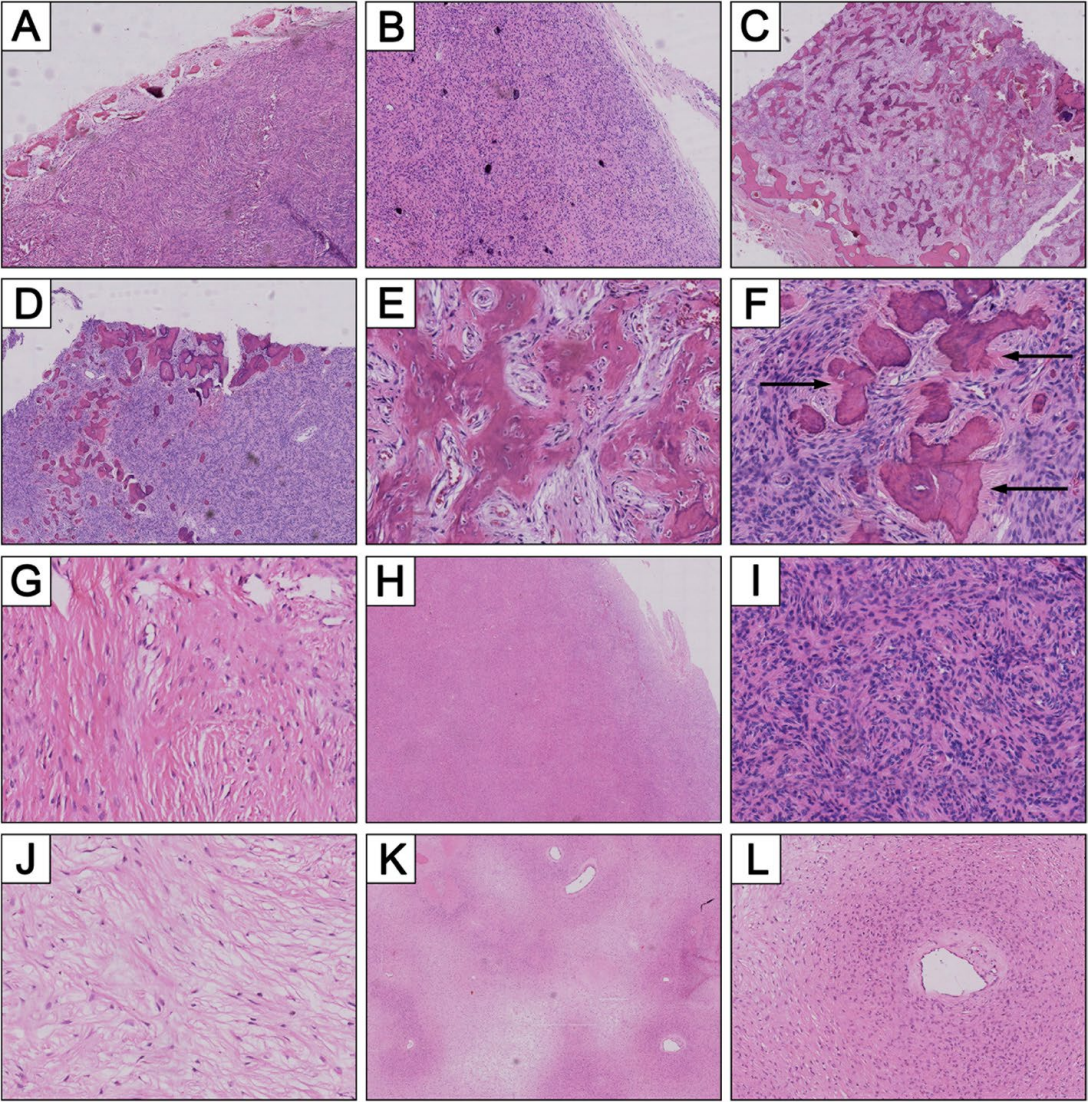
Representative pathological findings of OF in both patient II:2 and III:1. **A** The well-demarcated tumor showing hypercellularity component. **B** A partly encapsulated leision in which psammomatoid calcified spherules lying in hypercellular stroma. **C** A mineralization component of the lesion and a thin layer of fibrous tissue which separated from the surrounding normal bone. **D** Low power H&E image demonstrates interface between mineralized and non-mineralized tissue. **E** Cellular osteoid trabeculae formation lacking of typical osteoblastic rimming, mineralization often at the center of these trabeculae (medium-magnification photomicrograph of **C**). **F** A medium power view of **D** showing acellular basophilic cementum-like structures. The calcified tissue shows a distinctive “brush boarder” that interfaces with the surrounding stroma (arrows). **G** Hypocellular area with collagenized stroma cells. **H** Fibrous component with moderatecellular density, absence of calcifications completely (low-magnification image). **I** Dense fibrous cells are hyperchromatic and absence of mitosis. **J** Hypocellular area demonstrating loose edema-like stroma. Low-magnification **(K)** and high-magnification **(L)** photomicrographs showing a perivascular growth pattern (perivascular cells are relatively higher density than those of away from vessels)

All the jaw tumor samples of patients III:1 and II:2 were microscopically examined in our department, featured as varied amount of calcified substance lying in a variable density of fibrous stroma (Fig. 2). Mineralization was not homogeneous, displaying in the form of immature woven bone or of cementum-like round structures. With a careful correlation of radiologic characteristics and microscopic features (Fig. 1 and Fig. 2), we established the diagnosis of OF in both patients.

**Fig. 3:**
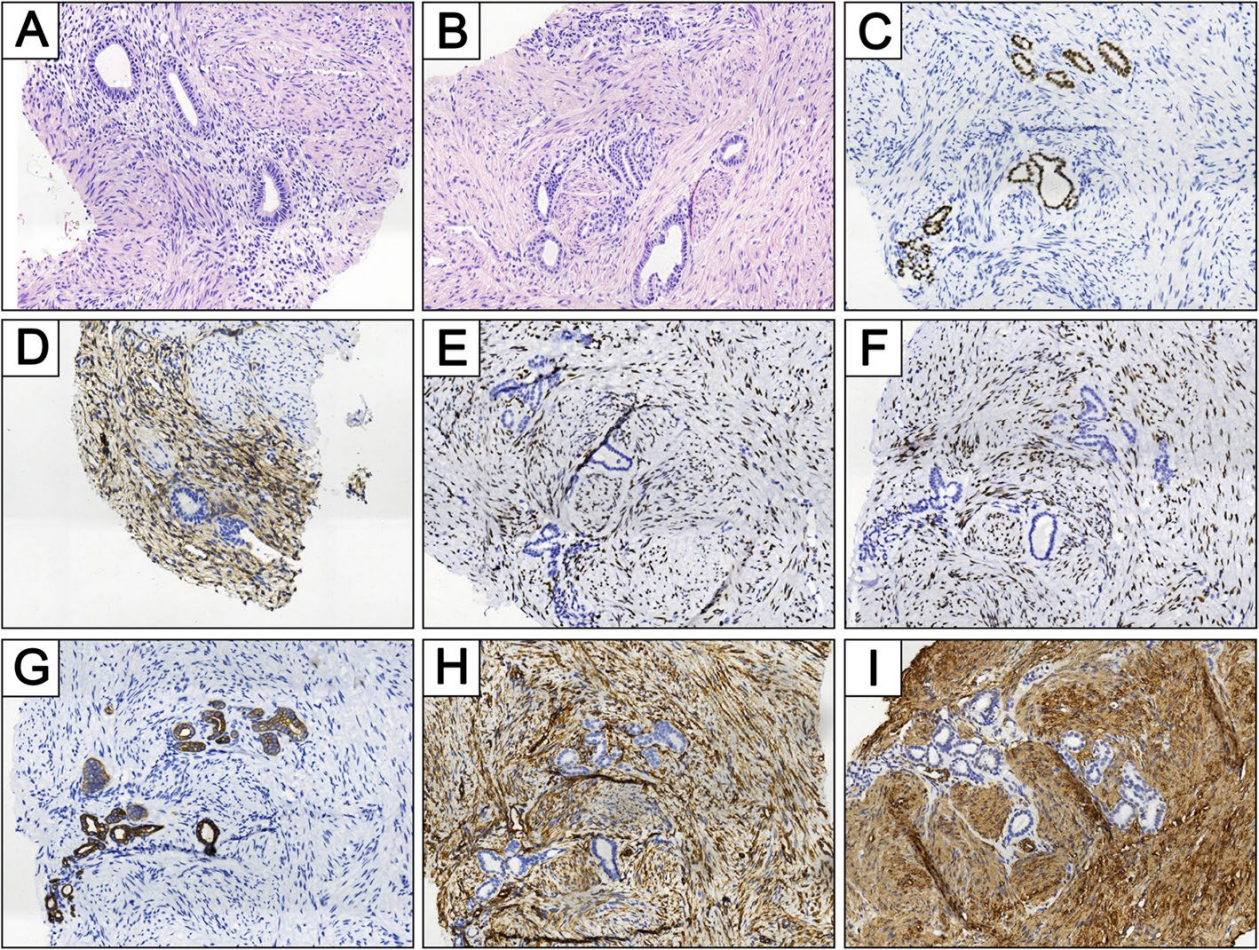
MEST histopathology and IHC. **A** and **B** Representative H&E stains of the patient III:1, transcutaneous puncture biopsy of the kidney, demonstrating a mixture of epithelial and mesenchymal components. Columnar epithelial cells formed tubular glands. The most of mesenchymal component is composed of uterine smooth muscle-like cells. A small componet of mesenchyme nearby the glands resembled endometrial stromal cells. **A** is reminiscent of uterus adenomyosis. Positive immunohistochemical staining of epithelial cells for **(C)** PAX8 and **(G)** CK. Mesenchymal cell is positive for **(E)** ER, **(F)** PR, **(H)** Vim and **(I)** SMA. **(D)** Endometrial stromal-like cells surrounding the glands exhibited expression of CD10

Morphologically, a core biopsy of the proband’s right kidney consisted of tubular epithelial components admixed with spindle cells with fascicles patterns. The most mesenchymal component resembled that of uterine smooth muscle (Fig. 3B). Fewer mesenchymal component which surrounded the epithelium consisted of cellular stroma with hyperchromatic nuclei and scant cytoplasm, resembling endometrial stromal cells (Fig. 3A). Tubular glands lined by columnar epithelium were similar to endometrioid-type glands. Immunohistochemically, the epithelial cells were positive for CK and PAX8; the stroma cells were positive for vimentin, ER, PR and SMA diffusely; and small focal endometrial stroma-like cells stained positively for CD10. Histologic classification of mixed epithelial and stromal tumor (MEST) were confirmed (Fig. 3).

**Fig. 4:**
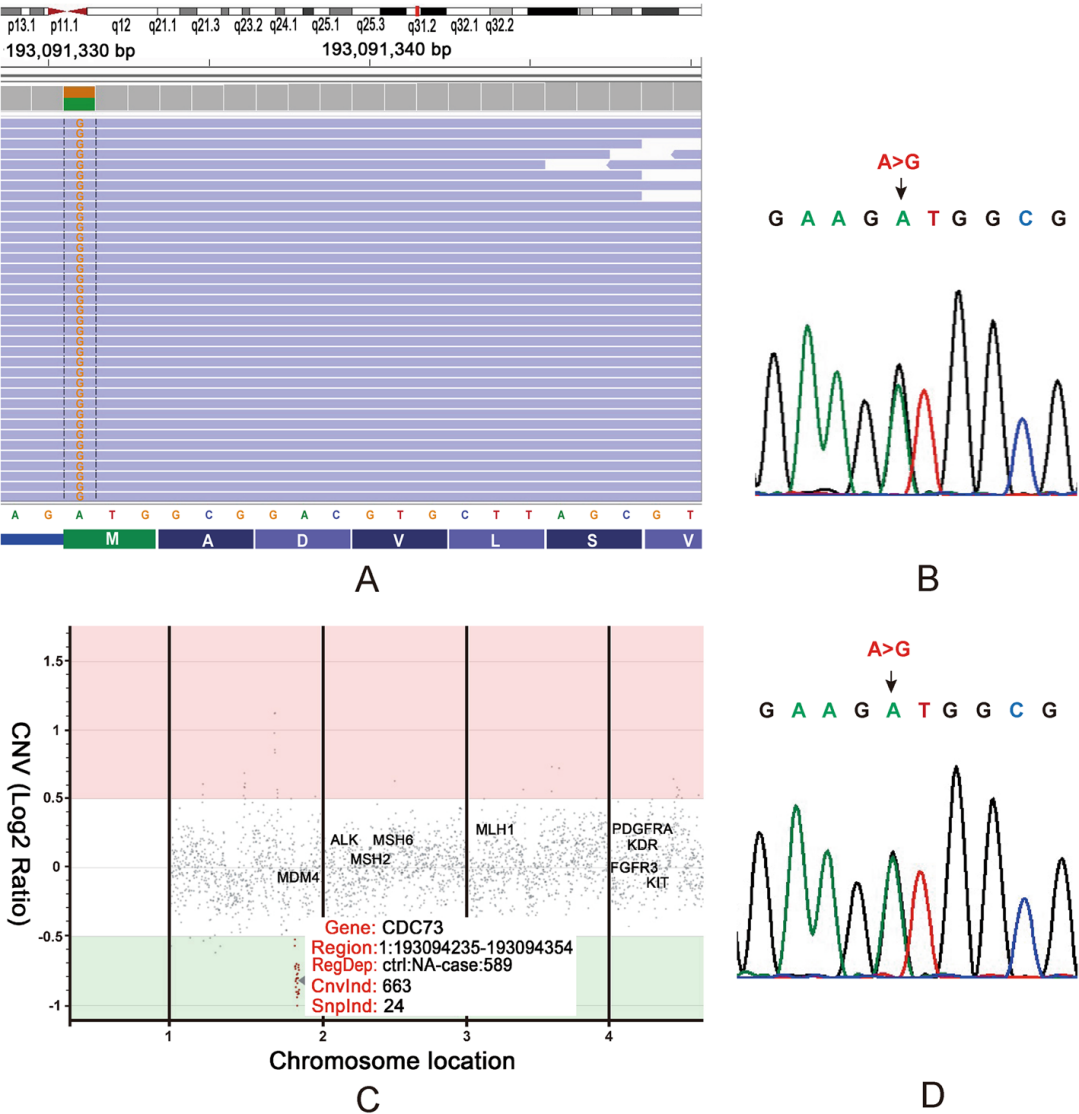
Gene testing of blood and jaw tumor DNA samples. **A** NGS testing of pan-tumor related 1021 genes demonstrating CDC73 germline point mutation (c.1A > G, p.Met1Val). **B** Chromatographs of CDC73 sequencing verify the heterozygous germline A to G change in the blood DNA from the patient III:1. **C** NGS visual image of the proband’s jaw OFs sample showing a heterozygous deletion of a whole region of CDC73 with CNV ratio 0.6 on chromosome 1. **D** Analysis of blood DNA from the patient II:2 demonstrated the same CDC73 germline mutation (c.1A > G)

In consideration of hereditary jaw disorders, the DNA samples extracted from tumor tissue of jaw and peripheral blood mononuclear cell (PBMC) of the patient III:1 were screened for genetic variation by next generation sequencing (NGS) based on a pan-tumor 1021-gene panel, including somatic and germline mutations. A germline CDC73 heterozygous mutation (c.1A > G, p.Met1Val) was identified (Fig. 4A). This mutation is recorded in ClinVar Database (https://www.ncbi.nlm.nih.gov/clinvar/), but not observed in Exome Aggregation Consortium (ExAC) Database (http://exac.broadinstitute.org/). At the same time, copy number loss at the CDC73 gene locus was identified in the jaw tumor DNA (Fig. 4C), indicating of a somatic second hit. Furthermore, the germline CDC73 mutation was verified by sanger DNA sequencing (Fig. 4B). In addition, the PBMC genomic DNA from family members of the proband were evaluated for this mutation. As was expected, the same germline CDC73 heterozygous mutation (c.1A > G) was observed in proband’s father, II:2 (Fig. 4D).

## Discussion and conclusions

We reported a family of 2 affected individuals, both lack of HPT or parathyroid tumor. This family hereditary syndrome was verified by genetic analysis, harboring the same CDC73 germline point mutation. In other reported index CDC73 germline mutation carriers, HPT was found in up to 95% of patients, caused mainly by a single benign parathyroid adenoma [2]. In nonindex CDC73 germline mutation carriers, a study has demonstrated HPT penetrance values raised with age (8, 53 and 75% at age 25, 50 and 70 respectively) [21]. Discrepancy of HPT penetrance rate between index and nonindex patients might imply that the onset of HPT is reminiscent of CDC73 related disorders. Conversely, missed diagnosis is easy to occur in patients without HPT. Although both patients had no clinical manifestation of HPT in our study, they had presented with recurrent and bilateral multiple OF in the jaw when they were preadolescent, which hinted the hereditary disorder. Genetic testing based on NGS was of great help to confirming a correct diagnosis of the rare disease. Recognizing HPT-JT syndrome is very important because of its high life-time risk of parathyroid carcinoma [11]. In contrast to other hereditary HPT (e.g. multiple endocrine neoplasia types 1, 2A and 4), parathyroid carcinoma (PC) is more frequent in patients with HPT-JT [2]. With regard to PC, the detection yield of CDC73 germline mutation is as high as 17 to 38%, especially higher in Chinese cohort [21–23]. Therefore, they should be on surveillance programs.

To our knowledge, the germline CDC73 heterozygous mutation (c.1A > G) was firstly reported here, which affects the initiator methionine of the CDC73 mRNA. The next in-frame initiating codon is located at codon 177. The expected rescue of translation at codon 177 may result in N-terminal truncation, missing the nuclear localization signal (residues 125–139) [24, 25], next resulting in loss-of-function of parafibromin. In combination with our reported kindred disorders, it was speculated this initiating codon mutation (c.1A > G, p.Met1Val) was pathogenic. However, more experiments are needed to prove that conclusively. Initiation codon variant of CDC73 has been published infrequently, the existing documents and records were also reviewed in Table. 1. Although all belong to initiation codon mutation, there was no clear mutation-specific phenotype.

**Table 1:** A review of initiation codon variants of CDC73 and related clinical presentation

References	Clinical presentation (number)				Germline mutation	Coding change
	HPT	JT	KL	UD		
[1]	4/4	2/4	4/4	0	c.3G > A	p.Met1Ile
Clinvar^a^	No record	Presence	Presence	No record	c.2 T > C	p.Met1Thr
[26]	0	0	3/3	2/3	c.3G > T	p.Met1Ile
Our report	0	2/2	1/2	1/2	c.1A > G	p.Met1Val

*HPT* Hyperparathyroidism, *JT* Jaw tumor, *KL* Kidney lesion, *UD* Uterine disease

Presence, presented with Nephroblastoma and Ossifying fibroma of the jaw, but the number is not referred to. No record; HPT or UD-related diseases is not recorded in databases

^a^National Center for Biotechnology Information. ClinVar; [VCV000521635.2], https://www.ncbi.nlm.nih.gov/clinvar/variation/VCV000521635.2

As a tumor suppressor gene, the first hit of CDC73 gene is heterozygous germline mutation, inherited from one of the parents or, in very rare cases, developed de novo at embryo level. In HPT-JT related tumor tissues, the second allele-inactivating mechanisms of CDC73 gene were loss of heterozygosity or epigenetic events, in agreement with Knudson’s two-hit hypothesis [1, 2, 27–30]. Interestingly, we firstly reported copy number loss of CDC73 in HPT-JT related jaw tumor, which may play as the somatic second hit. Regrettably, there were no sufficient kidney biopsy specimen for genetic testing after immunohistochemistry.

As a clinical manifestation of HPT-JT syndrome, OF of the jaw occur in 30% of HPT-JT patients. However, the penetrance rate of jaw is likely to be underestimated, because some patients in whom the OF develop before the HPT may not be correlated with HPT-JT. In HPT-JT patients with presentation of jaw tumor, 25% of the jaw tumor precede the development of HPT [31]. On the other hand, diagnosis of OF in the jaw is particularly challenging for the pathologist, and may be misdiagnosed as OD or FD. The three major forms of FOLs of the jaw are characterized histologically by a fibrous stroma with heterogeneous mineralized products [32]. In our reported patients, jaw lesions possessed the following characteristics: (1) early onset, multiple, bilateral and recurrent tumors; (2) well-defined and had a narrow uniform, partial or complete, radiolucent border representing soft tissue encapsulation, which is confirmed by pathology (Fig. 2A, B, C); (3) in contrast to old lesions, de novo jaw tumor in the right mandible displayed a lower density on CT scan and contained less or no mineralization histopathologically (Fig. 1B and Fig. 2H); (4) the amount and type of mineralization varied among tumors or even within the same tumor, the common type of mineralization are cementum and immature trabeculae. Need to add that, perivascular growth pattern of stroma cells enriched our pathological understanding (Fig. 2K, L), which was not reported by anyone else. As an important differential diagnosis, FD occurs in the first or second decade of life, may be polyostotic, and even be a part of MAS [18]. In MAS, FD can be accompanied by HPT which is a relatively rare symptom of MAS [33]. In this case, MAS is easily to be confused with HPT-JT. Radiographically, FD shows “ground glass” appearance, with ill-defined margin that transition and blend into the normal adjacent bone. Microscopically, FD shows bone with shapes similar to “alphabet soup or Chinese characters” and a general lack of osteoblastic rimming [17, 19]. The quantitative ratio of fibrous tissue to bony trabeculae seems rather stable in different zones of a single lesion (microscopic homogeneity) [34]. In genetics, the molecular basis of MAS is a postzygotic somatic mutation of GNAS gene. Moreover, most of sporadic FD lesions show missense mutations of GNAS, whereas no GNAS mutation is found in OF [35]. Successfully identifying OF have reminded us of HPT-JT, rather than MAS.

Besides OF, the proband had MEST and uterine disorders (leiomyoma and endometrial hyperplasia). HPT-JT related MEST is very limited [26, 36], our research has enriched related literature. A study of pathological characteristics of MEST have revealed that MEST was comprised of diverse epithelial and stromal elements [37]. In our report, heterogeneity of diverse elements was not shown completely due to limited biopsy specimen. However, cellular stroma showed smooth muscle differentiation and expression of ER and PR, epithelium component was negative for ER and PR, all of which were in accord with John N. Eble’s research [37]. MEST is most often seen in women and is associated with estrogen exposure. It occurs rarely in males and is sometimes associated with androgen deprivation [38]. Uterine disorders of female patients are common in HPT-JT [4]. It is well known that leiomyoma and endometrial hyperplasia of uterus are estrogen-dependent. Taking these into consideration, we suspect that estrogen plays an important role in the tumorigenesis of the proband. In terms of treatment, since vascular involvement and rarely malignant transformation of MEST, surgery is needed [39]. However, due to the risk of recurrent and/or denovo MEST in CDC73 mutation carriers, nephron-sparing surgery is proposed to preserve renal function [26]. In regard to treatment of uterine lesions, Wolff et al. described that aromatase inhibitor was effective in HPT-JT patients, rather than progestin [40].

## Abbreviations

HPT-JT: Hyperparathyroidism-Jaw Tumor
HPT: Hyperparathyroidism
OF: Ossifying fibroma
FOLs: Fibro-osseous lesions
OD: Osseous dysplasia
FD: Fibrous dysplasia
MAS: McCune-Albright Syndrome
NGS: Next generation sequencing
PC: Parathyroid carcinoma
MEST: Mixed epithelial and stromal tumor
